# Social media and the spread of misinformation: infectious and a threat to public health

**DOI:** 10.1093/heapro/daaf023

**Published:** 2025-03-31

**Authors:** Emily Denniss, Rebecca Lindberg

**Affiliations:** School of Exercise and Nutrition Sciences, Deakin University, 221 Burwood Hwy, Burwood, Victoria 3125, Australia; School of Health and Social Development, Deakin University, 221 Burwood Hwy, Burwood, Victoria 3125, Australia; Institute for Physical Activity and Nutrition, Deakin University, 75 Pigdons Road, Geelong, Victoria 3216, Australia; School of Exercise and Nutrition Sciences, Deakin University, 221 Burwood Hwy, Burwood, Victoria 3125, Australia; Institute for Physical Activity and Nutrition, Deakin University, 75 Pigdons Road, Geelong, Victoria 3216, Australia

**Keywords:** misinformation, social media, disinformation, health information, digital policy

## Abstract

Misinformation has been identified as a major threat to society and public health. Social media significantly contributes to the spread of misinformation and has a global reach. Health misinformation has a range of adverse outcomes, including influencing individuals’ decisions (e.g. choosing not to vaccinate), and the erosion of trust in authoritative institutions. There are many interrelated causes of the misinformation problem, including the ability of non-experts to rapidly post information, the influence of bots and social media algorithms. Equally, the global nature of social media, limited commitment for action from social media giants, and rapid technological advancements hamper progress for improving information quality and accuracy in this setting. In short, it is a problem that requires a constellation of synergistic actions aimed at social media users, content creators, companies, and governments. A public health approach to social media-based misinformation that includes tertiary, secondary, and primary prevention may help address immediate impacts, long-term consequences, and root causes of misinformation. Tertiary prevention to ‘treat’ this problem involves increased monitoring, misinformation debunking, and warning labels on social media posts that are at a high risk of containing misinformation. Secondary prevention strategies include nudging interventions (e.g. prompts about preventing misinformation that appear when sharing content) and education to build media and information literacy. Finally, there is an urgent need for primary prevention, including systems-level changes to address key mechanisms of misinformation and international law to regulate the social media industry. Anything less means misinformation—and its societal consequences—will continue to spread.

## Graphical Abstract

**Graphical Abstract:**
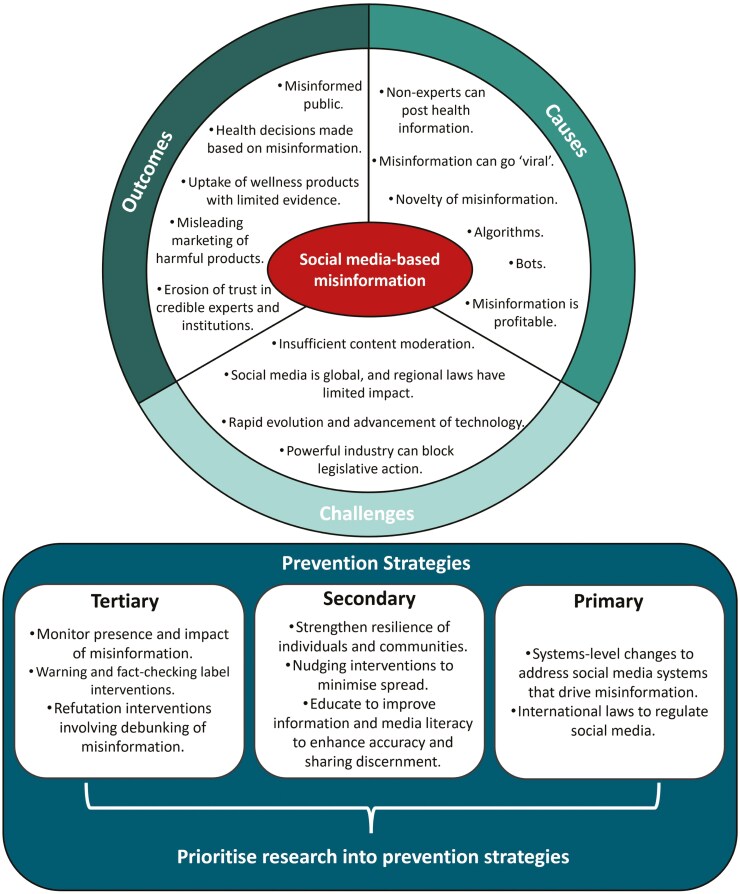

**Contribution to Health Promotion**

- Social media-based misinformation threatens public health through the provision of misleading information and undermines trust in credible experts and organizations.
- There are many interrelated causes of the misinformation problem and complex challenges for addressing it.
- A combination of complementary approaches to address misinformation and prevent its harms is required, informed by the World Health Organization competency framework for managing infodemics.
- We advocate for the uptake of widely recommended strategies, such as increased monitoring, misinformation debunking, and education interventions.
- We suggest international law as a potential strategy for bringing about systems-level changes to prevent misinformation.

## INTRODUCTION

We are living in the ‘post-truth era’. The World Economic Forum’s Global Risks Report identified misinformation and the technology that spreads it as a major global threat (World Economic Forum 2024). Furthermore, the World Health Organization (WHO) has made misinformation a priority area (WHO Regional Office for Europe 2022). Misinformation is defined by Treen et al. (2020) as ‘misleading information that is created and spread, regardless of whether there is an intent to deceive’, they also define disinformation as ‘[mis]information that is created and spread with intent to deceive’. Social media is now a useful tool for information dissemination in public health (De Vere Hunt and Linos 2022); however, it also contributes significantly to the spread of misinformation globally (Council of Canadian Academies [CCA] 2023). Social media-based misinformation is a complex problem with many consequences for public health (The Lancet 2025). This article will discuss the public health outcomes, causes, and challenges of misinformation. The evidence about intervention strategies is considered and using a public health prevention approach we suggest a combination of synergistic actions to address the immediate impacts, long-term consequences, and root causes of misinformation.

## PUBLIC HEALTH OUTCOMES OF MISINFORMATION VIA SOCIAL MEDIA

Health-related misinformation can mislead the public and thwart public health programmes. A large body of research has shown health misinformation, spanning a range of topics, including vaccines, infectious disease, nutrition, climate change, cancer, and smoking, is widely prevalent on major social media platforms (Wang et al. 2019, Treen et al. 2020, Suarez-Lledo and Alvarez-Galvez 2021, Denniss et al. 2023a). Even before the COVID-19 pandemic, there was an amplification of the ‘anti-vaxx’ movement, largely driven by the spread of misinformation, chiefly on social media (Hussain et al. 2018, Wang et al. 2019). Vaccine misinformation has led to reduced vaccination rates and outbreaks of disease, including measles in areas where elimination had been previously achieved (Hussain et al. 2018). The pandemic then brought vaccination to the forefront of public discourse. Global health misinformation networks published COVID-19 and vaccine misinformation, generating 3.8 billion views on Facebook in a 12-month period (Avaaz 2020). Misinformation and conspiracy theories contributed to COVID-19 vaccine hesitancy and caused increased fear and anxiety throughout the pandemic (Wilson and Wiysonge 2020, Rocha et al. 2023).

In addition, many so-called health and wellness influencers and brands use social media to spread misinformation for economic benefit (Lofft 2020, Baker 2022, CCA 2023). Recently, public health researchers have called for the social media industry to be recognized as a commercial determinant of health (CDoH), partly due to the financial incentives media giants receive to host misinformation on their platforms (Zenone et al. 2022). Marketing of products through sponsored advertising via payments to platforms and employment of influencers and brand presence is prolific across all major platforms, and marketing of health products, including supplements, weight loss, and fitness services, is particularly widespread (Lofft 2020, Denniss et al. 2023b). Research has shown wellness marketing content on social media often contains misinformation (Baker 2022, CCA 2023, Denniss et al. 2024). The largely unregulated social media environment means that influencers and brands can continue to monetize their content while breaching the platform’s community guidelines (Moran et al. 2024). Some health and wellness influencers have been known to cultivate alternative communities on social media and weaponize conspiracies, such as anti-vaxx misinformation, to sell wellness products (Baker 2022, Moran et al. 2024). These factors suggest that many social media users are exposed to misleading marketing content, which puts them at risk of purchasing health products and services with little to no evidence of efficacy, potentially leading to financial losses, minimal or no health benefits, and, in some instances, worse health outcomes.

Key industries whose systems, practices, and products are recognized as being CDoH also contribute to social media-based misinformation. The tobacco industry, which has historically misled the public about the harms of tobacco products, has marketed new nicotine products (e.g. e-cigarettes or ‘vapes’ and smokeless tobacco) through social media influencers, some of whom claim the products are harmless (Tan and Bigman 2020). Tobacco and other harmful industries, such as the suntanning industry, are increasingly presenting themselves as scientific authorities by employing disinformation tactics, including emphasizing areas of scientific uncertainty and conducting or commissioning research that serves their interests (Reed et al. 2021). Furthermore, health professionals with financial conflicts of interest due to direct and/or indirect funding from the industry have published misinformation on social media. For example, in the USA, dietitians and doctors with large followings have partnered with big food companies to promote consumption of discretionary foods as part of the ‘anti-diet’ movement and assert that consumption of artificial sweeteners is not linked to any health risks, contradicting WHO guidance (Chavkin et al. 2023, 2024). Experimental research has found that young adults who were exposed to misleading social media content about vapes exhibited more favourable attitudes towards vapes compared with a control, suggesting that misleading marketing may be influential and a tactic that can be exploited by harmful industries (Albarracin et al. 2018).

Finally, the circumstances described above contribute to the erosion of trust in health and science-related fields. Health misinformation is often divisive, and wellness influencers are known to undermine public health and science authorities (Baker 2022, CCA 2023, Moran et al., 2024). Such health-related misinformation contributes to polarization and the public’s mistrust in credible health experts and organizations (Penders 2018, CCA 2023, The Lancet 2025). Health professionals and experts with conflicts of interest, whether real or perceived, also contribute to the erosion of trust in public health (Penders et al. 2017). Many people instead trust alternative sources that spread misinformation and discredit legitimate health experts, creating a paradox: the mistrust fuelled by misinformation enables its continued spread (CCA 2023). This is problematic because mistrust has implications for the public’s adherence to evidence-based health advice, which can impact the health of individuals and populations (CCA 2023). Once trust has been eroded, it is difficult and time-consuming to restore (Penders 2018, Caulfield 2020, CCA 2023). Furthermore, the prevalence of misinformation and erosion of trust in public health may also serve the interests of harmful industries by undermining and distracting from the discussion of regulation and other measures.

## MECHANISMS THAT CAUSE MISINFORMATION

Social media environments are particularly conducive to the spread of misinformation, and there are several mechanisms that cause this. First, social platforms allow users to instantaneously publish on virtually any topic, regardless of their qualifications or information accuracy (Gajović and Svalastog 2016, Merchant and Asch 2018, World Economic Forum 2013). Platforms enable rapid sharing of content, and misinformation can ‘go viral’, cause harm, and change beliefs before it can be effectively corrected (World Economic Forum 2013, Merchant and Asch 2018, Vosoughi et al. 2018). Furthermore, research has shown that posts on X that contained misinformation received higher levels of engagement, spread more rapidly, and reached more users compared with posts that were truthful (Vosoughi et al. 2018). The same study also found that misinformation tended to be more novel, which is believed to be one reason it is spread more quickly than the truth (Vosoughi et al. 2018).

Second, social media algorithms contribute to the misinformation problem. Algorithms dictate content that appears in social media feeds based on users’ previous online behaviour (Pariser 2011, Zimmer et al. 2019). They are a tool to individualize feeds to deliver users content they are likely to find interesting and engage with so they spend more time using the platform (Zenone et al. 2022). These sophisticated algorithms use individuals’ social media data, including their social network, location, content they have viewed and engaged with, and content they have ignored, to predict what the user may prefer (Pariser 2011, Cohen 2018). Thus, social media users are shown an incomplete view of the scope of content published on a platform, whereby content that may challenge or oppose their beliefs is omitted (Pariser 2011, Cohen 2018). This can consolidate people’s ideologies or beliefs because they may not be aware that opposing information is hidden. In some instances, algorithms have delivered users content that is incrementally more subversive and divisive, leading to radicalization and extreme views (Ribeiro et al. 2020, Matthews 2022). In the context of misinformation, the algorithm continues to feed misinformation to users who engage with it, and these users are unlikely to be presented with information that discredits or corrects it. Social media algorithms give rise to echo chambers (i.e. communities of people with similar views) (Bessi et al. 2015, 2016, Zimmer et al. 2019, Cinelli et al. 2021), and social network modelling has shown that misinformation spreads more rapidly within these conditions (Törnberg 2018).

Third, internet robots or ‘bots’ are known to automate the publication of misinformation and disinformation on social media. Social bots imitate human users on social media and automatically post and reshare content, often relating to controversial topics within politics and health (Yuan et al. 2019, Himelein-Wachowiak et al. 2021). Bots can operate together in a coordinated manner in a ‘botnet’ (Abokhodair et al. 2015) and use tactics, including tagging and replying to influential accounts to increase their exposure and manipulate users to reshare misinformation (Shao et al. 2018). Investigations by journalists have revealed the existence of groups that coordinate disinformation campaigns using social bots, some of whom have reportedly interfered with elections in multiple regions (Kirchgaessner et al. 2023, Hendy 2024). Bots can be difficult for social media users, researchers, and even social media platforms to identify, making them hard to counteract (Hendy 2024). However, tools for bot detection, e.g. Botometer, have been able to detect bot activity, which suggests social media giants could be doing more to identify and remove bots (Indiana University Observatory on Social Media 2024).

Fourth, content creators and social media platforms are financially incentivized for publishing misinformation. Content creators earn income from social media through user views, promoting their own products, and by partnering with brands who pay them to market products, with brands paying more to creators with greater followings and engagement (Hua et al. 2022, Simonson and Williams 2024). Influencers can make more money by creating content that generates higher views and engagement, and misinformation is linked to greater views and engagement, particularly when it is novel (Vosoughi et al. 2018). There is a risk, therefore, that creators may be financially rewarded for posting health misinformation (Moran et al. 2024). Simultaneously, platforms make greater profits from users spending more time on social media through advertising revenue and generating more user data, which is sold (Zenone et al. 2022). Platforms leverage algorithms to keep people online for longer and continue to deliver misinformation to users who are likely to engage with it. Furthermore, misinformation in and of itself can generate revenue because people spend time viewing it (Vosoughi et al. 2018). This phenomenon has been evident in relation to COVID-19 and vaccination, whereby influencers leveraged vaccine and other health misinformation to profit from engagement and product sales (Moran et al. 2024); meanwhile, social media giants profited from people spending time viewing COVID-19 misinformation (Avaaz 2020). The situation in which both the creator and platform are financially incentivized to publish misinformation is a vicious cycle that allows misinformation to flourish.

Lastly, an interplay between human psychology and algorithms contributes to misinformation’s perpetuation (Rubin 2019). Most people are susceptible to confirmation bias, the inclination to believe information consistent with one’s beliefs (Nickerson 1998), and repetition bias, the tendency to believe information one has heard multiple times (Hassan and Barber 2021). Algorithms repetitively present users with information that aligns with their beliefs, meaning it is likely to be believable. Furthermore, people are generally information overloaded and time poor and are therefore unable to comprehensively fact-check or critically evaluate the plethora of information they are presented with online (Rubin 2019). Health misinformation can be difficult to detect, and many individuals have limited information, media, and health literacy, which makes it more difficult to discern accuracy (Rubin 2019, Australian Institute of Health and Welfare 2023). Humans also tend to exhibit truth bias, which makes them inclined to believe that the information they are presented with is true unless given significant reason to believe otherwise (Levine 2014, Rubin 2019). In combination, these factors contribute to humans’ susceptibility to misinformation, and research has shown that humans are more likely than bots to repost misinformation (Vosoughi et al. 2018).

## CHALLENGES FOR CURBING MISINFORMATION VIA SOCIAL MEDIA

Social media-based misinformation is a complex problem. There are numerous complicated challenges for addressing the mechanisms described above and the broader commercial and societal context within which this public health problem exists. One challenge is that social media operates globally, which creates complexity for legislation. Typically, legislative action is taken by a country or region (Centre for Digital Wellbeing 2021) and is therefore likely to have a limited impact. For example, an Australian regulatory body banned influencers from posting testimonials about therapeutic goods, including sunscreen and supplements (Therapeutic Goods Administration [TGA] 2022a), and fined individuals and businesses for posting inaccurate information about therapeutic goods (TGA 2021, 2022b). However, Australian social media users are likely to be exposed to material from international influencers who are not obligated to abide by Australian laws. Comprehensive monitoring of social media content for compliance with regulations is also difficult to achieve due to the volume of content posted on social media daily. Furthermore, this type of regulation only penalizes the entities responsible for publishing misleading information, and the platforms that facilitate misinformation face no recourse.

An additional challenge is that social media giants have shown insufficient commitment to content moderation and the removal of misinformation. Thus far, actions taken by platforms have been shown to be limited in their effectiveness. For example, the analysis of misinformation about the COVID-19 pandemic showed that only 16% of misinformation on Facebook carried a warning label (Avaaz 2020). A former Meta employee and whistleblower revealed research documents from Meta that indicate the company is aware that misinformation, divisive content, and hate speech on their platforms impact societies globally, and only a small proportion of this content is removed (Pelley 2021). Elon Musk, who acquired X in 2022, reduced the size of his content moderation team and has publicly voiced his aversion to content moderation (CBS News 2022). In February 2023, Musk posted on X, ‘All things in moderation, especially content moderation’ (Musk 2023). More recently, in January 2025, Mark Zuckerberg announced that Meta would be dropping their independent third-party fact-checkers, stating that they led to ‘too much censorship’ without effectively addressing misinformation (Watt et al. 2025), despite evidence that fact-checking can reduce belief in misinformation (Porter and Wood 2021). These examples demonstrate the lack of commitment to meaningful content moderation by major platforms, which may be due to the large investment that would be required and the loss of revenue from people viewing divisive content.

A further barrier is that technology and social media are continuously and rapidly evolving. Platforms can make seemingly instantaneous changes to their algorithm or interface that have significant implications for the functionality of the platform for millions of users. There has been a surge in artificial intelligence (AI) in recent years, much of which has been funded by tech giants, including Meta and Google (Buttazzo 2023). AI is predicted to reach human intelligence by 2030 and far exceed it in the years that follow (Buttazzo 2023), and social media platforms, including Instagram, LinkedIn, and Snapchat, have embedded AI tools into their platforms. Many implications of technological innovations are not known until after they have been rolled out, and it can then be difficult to put the genie back into the bottle. These rapid and unprecedented technological advancements make it difficult to develop and implement timely and effective policies. Meta CEO Mark Zuckerberg’s motto, ‘move fast and break things’ (Hamilton 2022), suggests big players in the industry not only understand but also take advantage of this.

Finally, social media companies are powerful corporate entities with extensive resources (Rabb 2018). Historically, these companies have circumvented consequences for unethical practice with relative ease (Newcomb 2018, McCallum 2022). In 2023, Meta, YouTube, and TikTok’s revenue totalled $134 billion, $32 billion, and $120 billion USD, respectively (Statista 2024a, 2024b, WARC 2024). Meta’s responses to privacy breaches on Facebook have historically involved apologies to users but limited changes to policy or operations (Newcomb 2018). After the 2018 Cambridge Analytica scandal, whereby 87 million Facebook users’ private data was harvested from Facebook and used for political advertising, Meta agreed to pay a $725 million USD settlement, a small percentage of their annual revenue (McCallum 2022). Since the scandal, Meta has continued to generate massive revenue and maintain a large user base. Changes to legislation that impact social media have resulted in backlash. In 2021, the Australian government passed a law that mandated social platforms pay news media outlets for publishing their articles (Meade 2021). Facebook reacted by blocking Australian users from accessing news content, which also blocked the pages of Australian health and emergency services, resulting in a key communication channel being disrupted. Meta leveraged this to negotiate changes to the legislation before unblocking pages and news content (Meade 2021). This example demonstrates the power that social media companies wield to block government actions and the impact their backlash can have on the people and organizations relying on their services.

## A WAY FORWARD: STRATEGIES FOR MISINFORMATION PREVENTION

Thus far, regulations and voluntary actions from social media companies have had a limited impact in addressing the misinformation problem, and urgent action is required. As has been seen with the regulation of other harmful industries, such as the tobacco industry, curbing the activities of social media companies is likely to take time, be fiercely opposed, and be difficult to implement (Daube and Maddox 2021). The literature suggests that complex problems are not ‘solved’ *per se* but can be managed and addressed through a range of incremental and complementary approaches (Head 2019). Therefore, a combination of priority strategies is suggested below. In public health, there is an emphasis on holistic approaches that involve tertiary, secondary, and primary prevention to address the immediate acute impacts, long-term consequences, and root causes of health issues (Goldsteen et al. 2010). Social media-based misinformation is a public health issue with immediate impacts (e.g. a misinformed public), long-term consequences (e.g. eroded trust in science and public health), and complex root causes (e.g. algorithms, bots, and financial interests). As such, a public health prevention approach to managing and addressing misinformation may be beneficial, and a combination of tertiary, secondary, and primary prevention strategies is presented.

### Tertiary prevention

Misinformation is widespread on social media, and tertiary prevention strategies to respond to and ‘treat’ the problem are required. The WHO competency framework for managing infodemics provides guidance for public health organizations in responding to misinformation. Whilst it was designed in the context of public health emergencies, many of the strategies remain relevant. The WHO framework has four domains, three of which relate to tertiary prevention. First, measure and monitor the impact of misinformation; second, determine how it is spread; and third, intervene to minimize consequences (Rubinelli et al. 2022). Interventions involving the inclusion of warning, fact-checking, and source-credibility labels on high-risk information on social media are effective strategies for minimizing harm (Kozyreva et al. 2024). Specifically, warning labels are used to caution users that a post is likely to contain misinformation, and fact-checking labels specify that a post contains false or partially false information. There is evidence that warning and fact-checking labels can improve accuracy, discernment, and sharing intentions (Kozyreva et al. 2024). During the COVID-19 pandemic, there was pressure on social media giants to prevent misinformation, and major platforms (e.g. Facebook and YouTube) placed warning labels that provided links to credible information (e.g. WHO website) on posts that discussed COVID-19 (Warnke et al. 2024). Advocates should put pressure on social media giants to embed warning and fact-checking labels in their platforms and provide access to their application programming interfaces to allow governments and public health organizations to more easily and comprehensively monitor misinformation trends.

Refutation interventions involving debunking of misinformation, whereby corrective information is provided, are an evidence-based strategy that has been shown to reduce belief in rumours and misinformation (Danielson et al. 2024, Kozyreva et al. 2024). While there has been concern about the possibility of debunking to backfire and reinforce misconceptions, a recent meta-analysis found no evidence of a backfiring effect and recommended the refutation of misinformation is published online (Danielson et al. 2024). Public health advocates and the broader health community, including medical professionals, academics, scientists, and public health organizations, have an important role to play in refuting misinformation and promoting evidence-based information in both online and offline settings, and this has been encouraged by others in the field (Ecker et al. 2024). While doctors and other health professionals have been seen to contribute to misinformation in some instances, they are generally well trusted by the community and can contribute to misinformation refutation in clinical and community settings, as was seen during the COVID-19 pandemic in rural areas of the USA (Howard 2021, Mainous et al. 2024).

### Secondary prevention

The final domain of the WHO framework relates to secondary prevention and involves strengthening the resilience of individuals and communities to misinformation (Rubinelli et al. 2022). There is a growing body of evidence investigating the impact of interventions on misinformation detection and reducing the sharing of misinformation (Kozyreva et al. 2024). ‘Nudging’ or prompting strategies have been used as components of comprehensive approaches to health promotion for many years, and research has shown that nudging can improve sharing intentions and discernment (Kozyreva et al. 2024). Examples of nudging include messages that remind people about the harms of misinformation or prompts to read an article in full and evaluate the accuracy of the headline before sharing (Kozyreva et al. 2024). Previously, X (then Twitter) embedded a ‘read before you retweet’ nudge in the platform that reminded users to read articles when reposting links that they had not clicked on (Verge 2020). Similar nudges could be embedded in social media platforms as a strategy to minimize the spread of misinformation.

Educational interventions to improve digital and media literacy have also shown promise in improving individuals’ accuracy, credibility, and sharing discernment (Kozyreva et al. 2024). Digital and media literacy skills are now vital life skills and competencies that are required at every life stage and within almost every vocation. School curricula for children are an obvious setting, and evidence-based programs show effectiveness (Dumitru et al. 2022). The need for information and media literacy education has been echoed by the WHO and others in the field (Rubin 2019, WHO Regional Office for Europe 2022), and strengthening the information and media literacy of populations is a priority area for reducing the spread and impact of misinformation. There is evidence that when the companies or industries involved in a public health problem contribute to education programs, the educative content has been skewed, e.g. stealth marketing of food brands in children’s food and nutrition education in schools (Wilkinson 2024) and industry-funded youth education programs about alcohol, tobacco and gambling that serve industry interests (Sebrié and Glantz 2007, van Schalkwyk et al. 2022a, Van Schalkwyk et al. 2022b). Therefore, education programs should be developed and run by governments, independent health agencies or other groups without conflicts of interest.

### Primary prevention

The adage ‘a lie can travel halfway around the world before the truth can get its boots on’ speaks to the need for primary prevention of misinformation. It is acknowledged in the literature that in addition to individual- and community-level interventions, systems-level changes are required to address the misinformation problem (Kozyreva et al. 2024). The current social media system not only facilitates the publication and spread of misinformation but also fuels and generates profits from it. Historically, voluntary corporate social responsibility actions by commercial actors to promote public health or prevent harm have been shown to hinder or delay meaningful and legitimate actions (Madureira Lima and Galea 2018, De Lacy-Vawdon and Livingstone 2020). Due to the highly profitable nature of social media, impactful voluntary action is unlikely; therefore, greater regulation is necessary. Many governments have policies that aim to regulate various facets of social media and minimize misinformation, including the European Union’s Digital Services Act, which came into effect throughout 2023–2024 (Centre for Digital Wellbeing 2021, European Union 2022). The Australian government recently proposed new laws to penalize social media companies for mis- and disinformation on their platforms; however, this was met with a mix of support and criticism and did not pass in the Senate (Rownland 2024, Speers and Truu 2024). Despite the challenges that exist, it is clear there is potential and an appetite in some jurisdictions for greater regulation.

The global nature of social media is a barrier to policy and legislative action. In an increasingly globalized world, the need for global health law to address widespread public health problems has been recognized, and treaty law is considered a promising tool to facilitate cooperation between states to achieve public health objectives that cannot be adequately addressed through regional or domestic laws and policies (Taylor 2017). One example is the WHO Framework Convention on Tobacco Control (FCTC), which is a treaty subject to international law that came into force in 2005 as a means for countries to cooperate to prevent the harms of tobacco (McInerney 2019). States that signed the FCTC agreed to actions including increased taxes, banning advertising, and regulating the content of tobacco products. Analysis of the FCTC found evidence that it had positively impacted tobacco control, in part due to its status as an international legal obligation (McInerney 2019). Links have been drawn between social media and tobacco because both are purposefully addictive and sold by powerful profit-driven companies (MacBride 2018, Holly et al. 2024). Therefore, the success of the FCTC in addressing the impacts of tobacco suggests that international law to regulate social media may be a promising strategy to address misinformation. Regulatory action via treaty could include mandating greater content moderation, removal of bots, and changes to algorithms to present users with a balanced view of content and show results from credible authorities when platforms’ search engines are used, similar to action taken by Pinterest for vaccine-related searches (Ozoma 2019).

It is important to note that international agreement on treaty law will be difficult to achieve. Regulation of harmful industries is challenging to implement at the national level, and these challenges are likely to be magnified at the international level due to diverging national interests (Taylor 2017). Negotiating international law is complex, which has historically made commitment and implementation by member states a time-consuming process (Taylor 2017). Furthermore, harmful industries have learned from the tobacco experience and use innovative tactics to avoid regulation and protect their profits (Thomas et al. 2024). It is therefore important that other avenues of legislation and systems-level change are also explored and targeted. To the knowledge of the authors, most misinformation research has focused on tertiary and secondary prevention and the impact of primary prevention; systems-level strategies for addressing misinformation are an important research gap that should be addressed to inform future policies and interventions.

## CONCLUSION

In conclusion, a combination of synergistic primary, secondary, and tertiary prevention strategies is required to address the public health impacts of social media-based misinformation. This perspective piece advocates for the use of the WHO framework for managing infodemics in conjunction with established and well-evidenced actions, such as monitoring, debunking, warning labels, and education. We contribute to the field by suggesting international treaty law as a primary prevention strategy to bring about the systems-level changes required to address the mechanisms and root causes of misinformation. International regulation and systems-level changes may also serve to address the broader health and societal impacts of social media, such as its purposefully addictive features. Meaningful cooperation from social media giants in all levels of prevention of misinformation would lead to a greater impact. Wherever possible, public health practitioners, researchers, organizations, and the broader health community should advocate for greater action from the social media industry to address misinformation. Finally, primary prevention and systems-level strategies to address misinformation should be a research priority area.

## Data Availability

No new data were generated or analysed in support of this research.
